# Identification of Sperm-Binding Sites in the N-Terminal Domain of Bovine Egg Coat Glycoprotein ZP4

**DOI:** 10.3390/ijms23020762

**Published:** 2022-01-11

**Authors:** Kamila Dilimulati, Misaki Orita, Yoshiki Yonahara, Fabiana Lica Imai, Naoto Yonezawa

**Affiliations:** 1 Department of Chemistry, Graduate School of Science, Chiba University, Chiba 263-8522, Japan; kamila221@hotmail.com (K.D.); mo311.pepupimo@ezweb.ne.jp (M.O.); licaimai@hotmail.com (F.L.I.); 2 Department of Chemistry, Faculty of Science, Chiba University, Chiba 263-8522, Japan; y_yoshiki@icloud.com

**Keywords:** zona pellucida, sperm-binding sites, fertilisation, glycoprotein, baculovirus

## Abstract

The species-selective interaction between sperm and egg at the beginning of mammalian fertilisation is partly mediated by a transparent envelope called the zona pellucida (ZP). The ZP is composed of three or four glycoproteins (ZP1–ZP4). The functions of the three proteins present in mice (ZP1–ZP3) have been extensively studied. However, the biological role of ZP4, which was found in all other mammals studied so far, has remained largely unknown. Previously, by developing a solid support assay system, we showed that ZP4 exhibits sperm-binding activity in bovines and the N-terminal domain of bovine ZP4 (bZP4 ZP-N1 domain) is a sperm-binding region. Here, we show that bovine sperm bind to the bZP4 ZP-N1 domain in a species-selective manner and that N-glycosylation is not required for sperm-binding activity. Moreover, we identified three sites involved in sperm binding (site I: from Gln-41 to Pro-46, site II: from Leu-65 to Ser-68 and site III: from Thr-108 to Ile-123) in the bZP4 ZP-N1 domain using chimeric bovine/porcine and bovine/human ZP4 recombinant proteins. These results provide in vitro experimental evidence for the role of the bZP4 ZP-N1 domain in mediating sperm binding to the ZP.

## 1. Introduction

The zona pellucida (ZP), a transparent coat surrounding mammalian oocytes, plays important roles in oogenesis, species-selective sperm recognition, blocking polyspermy and early embryonic development [1,2]. Mammalian fertilisation requires several steps. In most species, capacitated sperm bearing an intact acrosome binds to the ZP (primary sperm-ZP binding). After binding, the sperm undergoes the acrosome reaction, which reinforces the binding to the ZP (secondary sperm-ZP binding). The acrosome-reacted sperm then reaches the perivitelline space and binds to the oolemma. Afterwards, gamete membrane fusion occurs [3,4].

The ZP contains three or four glycoproteins depending on the species. There are four ZP glycoproteins in humans (hZP1, hZP2, hZP3 and hZP4) [5], whereas three ZP glycoproteins exist in pigs (pZP2, pZP3 and pZP4), bovines (bZP2, bZP3 and bZP4) [6] and in mice (mZP1, mZP2 and mZP3) [7]. In mice and humans, the N-terminal domain of ZP2 binds sperm [8,9]. In pigs, ZP4 is responsible for the sperm-binding activity of the ZP3/ZP4 complex [10]. Recently, we showed that ZP4 binds to sperm in bovines [11]. The species difference in sperm-binding ZP glycoproteins suggests that sperm-ZP binding mechanisms are different amongst mammals.

**Figure 1:**
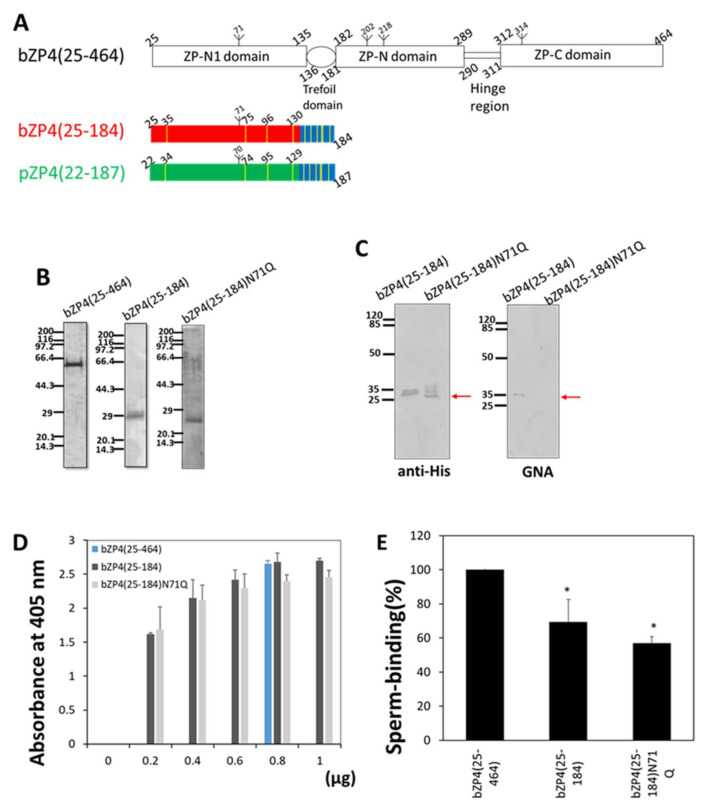
Effect of N-glycosylation site mutation of the N-terminal domain of bovine ZP4 on the sperm-binding activity of the domain. (**A**) Domain architecture of mature bovine (b)ZP4(25–464) polypeptide, and a schematic representation of the truncated bZP4(25–184) consisting of the ZP-N1 domain (red) and trefoil domain (blue) and its corresponding porcine (p)ZP4(22–187) (ZP-N1 domain, green; trefoil domain, blue) counterpart examined in this study. Inverted tripods mark potential N-glycosylation sites, and cysteine residues are highlighted in yellow bars in bZP4(25–184) and pZP4(22–187). (**B**) Sodium dodecyl sulphate polyacrylamide gel electrophoresis (SDS-PAGE) analysis of the bZP4(25–464), bZP4(25–184) and N-glycosylation site mutant, bZP4(25–184) N71Q. These proteins were expressed in Sf9 cells and purified by TALON resin specific to His-tag. The SDS-PAGE gels were silver-stained. Molecular mass standards (kDa) are indicated on the left side of each gel. (**C**) Western blot analyses of bZP4(25–184) and bZP4(25–184) N71Q. Both proteins were detected by immunoblotting with anti-His antibody (anti-His), while only bZP4(25–184) was detected by *Galanthus nivalis* agglutinin (GNA). The objective bands are indicated by arrows. Molecular mass standards (kDa) are indicated on the left side of each panel. (**D**) Adsorption of bZP4(25–184) and bZP4(25–184) N71Q to plastic wells. The amount of protein necessary for adsorption saturation was examined by detecting the adsorbed protein with an antibody specific to the N-terminal His-tag. The amount of bZP4(25–184) (dark gray) or bZP4(25–184) N71Q (light gray) added to one plastic well is indicated under each bar. The experiment was performed three times and the average ± standard deviation (SD) of absorbance at 405 nm is shown. Absorbances of these proteins reached the same level as that of bZP4(25–464) at 0.8 µg (blue). (**E**) Comparison of sperm-binding activity between bZP4(25–184) and bZP4(25–184) N71Q. Plastic wells were coated with the ZP4 proteins (0.8 μg for each protein) indicated in the graph. The number of sperm bound to wells coated with bZP4(25–464) varied from 35 to 86 but was designated as 100%. Assays were repeated five times. Data are presented as the mean ± SD. There was no significant difference between the activities of bZP4(25–184) and bZP4(25–184) N71Q (* *p* > 0.05).

All ZP proteins have a ZP module that is important for the formation of ZP filaments. The ZP module consists of two structurally related immunoglobulin (Ig)-like domains (ZP-N and ZP-C) connected to each other by a short, flexible hinge region (see Figure 1A) [12,13]. ZP proteins are highly heterogeneous because asparagine (N-linked glycosylated) and serine/threonine (O-linked glycosylated) residues are glycosylated to different extents. In many species, the glycans on ZP proteins are involved in the sperm-ZP binding in a species-selective manner [14,15,16].

In bovines and pigs, ZP2, ZP3 and ZP4 amino acid sequences are well conserved having 77%, 85% and 75% identity, respectively [17]. The estimated protein molar ratio of ZP2/ZP3/ZP4 for native bovine ZP is 1:2:1 [18], whereas it is 1:6:6 for native porcine ZP [19], suggesting that the bovine and porcine ZP filamentous structures are different. Interestingly, ZP4 is responsible for ZP sperm-binding activity in bovines and pigs [10,11], suggesting that the sperm-ZP interacting mechanisms between these two species are conserved to some extent. 

By developing a solid support assay, we recently demonstrated the sperm-binding activity of two regions of bZP4: the region extending from Lys-25 to Asp-136, which almost corresponds to the N-terminal ZP-N1 domain, and the one extending from Ser-290 to Lys-340, which consists of the flexible hinge region and the N-terminal part of the ZP-C domain (see Figure 1A) [11]. The sperm-binding activity of bZP4(25–136) is a little higher than that of bZP4(290–340). It was hypothesised that the bZP4(290–340) interaction with bZP3 enhances its sperm-binding activity [11]. Although these results document the dependency of sperm recognition on bZP4(25–136), it is still unknown which region of the bZP4(25–136) domain is involved in the sperm-binding activity. In the mouse ZP2, the region extending from residues 52 to 83 in the N-terminal domain includes the sperm-binding site, and the N-glycosylation at the single putative site is not necessary for the sperm-binding activity [9,20]. There are four potential N-glycosylation sites in bZP4: at Asn-71, -202, -218, and -314. The Asn-71 site is located in bZP4(25–136). The role of Asn-71 N-glycosylation in the sperm-binding activity of bZP4(25–136) remains to be determined.

In this study, to precisely identify the sperm-binding sites of bZP4(25–136), we analysed the involvement of N-glycosylation at the Asn-71 site in the sperm-binding activity and analysed the sperm-binding sites in bZP4(25–136) using a solid support assay. 

## 2. Results

### 2.1. N-Glycosylation Is Not Required for bZP4(25–184) Sperm-Binding Activity 

Recently, we showed that bZP4 has a predominant binding activity for bovine sperm in a solid support assay [11]. The bZP4 polypeptide expressed in Sf9 cells included residues Lys-25 to Arg-464 and consisted of five regions: N-terminal ZP-N-like domain (Lys-25 to Pro-135, ZP-N1), trefoil domain (Asp-136 to Tyr-181), ZP-N domain (Gly-182 to Ala-289, ZP-N), hinge region (Ser-290 to Gln-311) and ZP-C domain (Pro-312 to Arg-464, ZP-C) (Figure 1A). The sperm-binding activity of bZP4(25–184) was shown to be similar to that of bZP4(25–136), indicating that the trefoil domain is dispensable for the sperm-binding activity [11]. The purification yield of bZP4(25–184) was a little higher than that of bZP4(25–136). Therefore, bZP4(25–184) was chosen as a starting material in this study. 

Previous studies suggested that α-mannose (Man) residues at the nonreducing termini of high-Man-type N-linked chains of bovine ZP are essential for sperm-binding [21,22,23]. Recombinant bZP4 expressed in Sf9 cells has pauci-Man-type N-glycans with α-Man residues at the nonreducing termini [17]. Additionally, there is a potential N-glycosylation site at Asn-71 of bZP4(25–184) (Figure 1A). Since recombinant bZP4 was not detected by *Amaranthus caudatus* agglutinin, the population of recombinant bZP4 possessing O-linked chains was low [17]. To investigate the role of N-glycosylation on bZP4(25–184) in sperm binding, Asn-71 was mutated to Gln. The bZP4(25–184) N71Q mutant was expressed in Sf9 cells and purified to near homogeneity (Figure 1B). The mutation of the N-glycosylated Asn-71 residue was confirmed, as *Galanthus nivalis* agglutinin (GNA) failed to recognise bZP4(25–184) N71Q. In contrast, bZP4(25–184) was recognised by GNA, indicating that the Asn-71 residue was N-glycosylated (Figure 1C). The sperm-binding activity of the recombinant proteins was examined by adsorbing them to plastic wells. We first assessed the amount of bZP4(25–184) and bZP4(25–184) N71Q required to saturate the adsorption of the proteins to the plastic wells and found that 0.8 μg of protein was enough for saturation (Figure 1D). The number of bovine sperm bound to the wells coated with bZP4(25–184) was 40% lower than that measured in bZP4(25–464)-coated wells, as reported previously (Figure 1E) [11]. The number of bovine sperm bound to wells coated with bZP4(25–184) N71Q was not significantly different from that obtained with bZP4(25–184) coating (Figure 1E), indicating that N-glycosylation is not necessary for bZP4(25–184) sperm-binding activity.

### 2.2. Bovine Sperm Recognise bZP4(25–184) but not pZP4(22–187) in the Solid Support Assay

**Figure 2:**
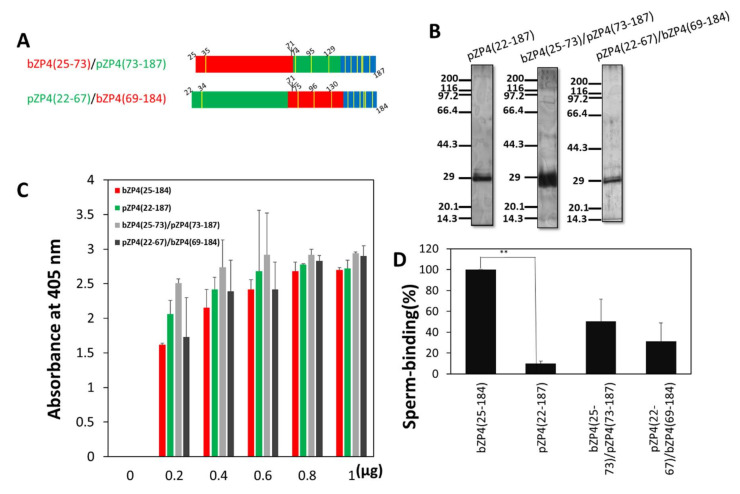
Two regions in bZP4(25–184) are involved in its sperm-binding activity. (**A**) Schematic diagrams of chimeric proteins between the N-terminal ZP-N1 domains of bZP4 (red) and porcine (p)ZP4 (green). Trefoil domains are shown in blue and Cys residues are in yellow bars. (**B**) SDS-PAGE of pZP4(22–187) and bovine/porcine and porcine/bovine chimeric fragments. pZP4(22–187), bZP4(25–73)/pZP4(73–197), and pZP4(22–67)/bZP4(69–184) were expressed in Sf9 cells and purified by TALON resin specific to His-tag. Gels were silver-stained. Molecular mass standards (kDa) are indicated on the left side of each panel. (**C**) Adsorption of bZP4(25–184) (red), pZP4(22–187) (green), bZP4(25–73)/pZP4(73–187) (light gray) and pZP4(22–67)/bZP4(69–184) (dark gray) to plastic wells. The amount of each recombinant ZP4 protein added to one plastic well is indicated under each group of bars. The amounts of proteins necessary for adsorption saturation were examined by detecting the adsorbed proteins with an antibody specific to the N-terminal His-tag. The experiment was performed three times and the average ± standard deviation (SD) of absorbance at 405 nm is shown. (**D**) Sperm-binding activity of the four recombinant ZP4 proteins shown in (**C**). Plastic wells were coated with each of the proteins (0.8 μg for each protein). The number of sperm bound to the wells coated with bZP4(25–184) varied from 33 to 65 but was designated as 100% for each experiment. Assays were repeated five times. Data are presented as the mean ± SD, with statistical significance between bZP4(25–184) and pZP4(22–187) indicated as *p* < 0.01 (**) on the line connecting the two bars. There was no significant difference between bZP4(25–73)/pZP4(73–187) and pZP4(22–67)/bZP4(69–184).

Next, we determined whether bovine sperm recognise pZP4(22–187) as it is similar to bZP4(25–184) (Figure 1A and Figure S1). Both fragments were expressed in Sf9 cells and purified to near homogeneity, as revealed by SDS-PAGE (Figure 1B and Figure 2B). The amount of pZP4(22–187) required to saturate the adsorption of the protein to the plastic wells was 0.8 μg, which was the same as that of bZP4(25–184) (Figure 2C) [11]. The number of bovine sperm bound to wells coated with pZP4(22–187) was significantly decreased compared with the number bound to bZP4(25–184)-coated wells (Figure 2D). This result indicated that bovine sperm recognised bZP4(25–184) but not pZP4(22–187).

### 2.3. Three bZP4(25–135) Regions Are Involved in Bovine Sperm Recognition

#### 2.3.1. Both N-Terminal and C-Terminal Halves of the ZP-N1 Domain of bZP4 Are Involved in Sperm Binding 

The amino acid sequences of bZP4 and pZP4 share 75% identity [17]. The bovine and porcine N-terminal ZP-N1 domains share 58% identity, whereas 88% identity was found between the bovine and porcine trefoil domains (Figure S1). Since the N-glycosylation was not essential for sperm binding and since we previously showed that O-glycosylation was not detected [17], the species-selectivity was most likely mediated by residues that were not identical in bZP4(25–135) and pZP4(22–136). 

The translational Met was numbered as 1 for the bZP4, pZP4 and hZP4 fragments. There were gaps in the bovine, porcine and human amino acid sequence alignment (Figure S1). Therefore, the numbering of corresponding residues did not match amongst the three fragments as shown below.

Since we could not make deletional mutants of bZP4(25–135) because of the disulphide bonds, our strategy followed that used to identify sperm-binding sites in the hZP2 N-terminal domain [9]. Two chimeric fragments were prepared (Figure 2A): bZP4(25–73)/pZP4(73–187) in which the bovine sequence extending from Asp-74 to Thr-184 of bZP4(25–184) was replaced by the porcine sequence extending from Gly-73 to Thr-187, and pZP4(22–67)/bZP4(69–184) in which the porcine sequence extending from Leu-68 to Thr-187 of pZP4(22–187) was replaced by the bovine sequence extending from Leu-69 to Thr-184 (Figure 2A). These chimeric fragments were expressed in Sf9 cells and purified to near homogeneity, as shown by SDS-PAGE (Figure 2B). The amount of protein required for saturation of the adsorption on plastic wells was 0.8 μg for both proteins (Figure 2C). The number of bovine sperm bound to wells coated with either bZP4(25–73)/pZP4(73–187) or pZP4(22–67)/bZP4(69–184) was reduced by about 50% compared with that bound to bZP4(25–184)-coated wells (Figure 2D). Interestingly, there was no significant difference in the sperm-binding activities of bZP4(25–73)/pZP4(73–187) and pZP4(22–67)/bZP4(69–184) (Figure 2D), suggesting that both regions (residues 25 to 73 and residues 74 to 184) were involved in the sperm-binding activity.

#### 2.3.2. Identification of Sperm-Binding Sites in the N-Terminal Half of the ZP-N1 Domain

**Figure 3:**
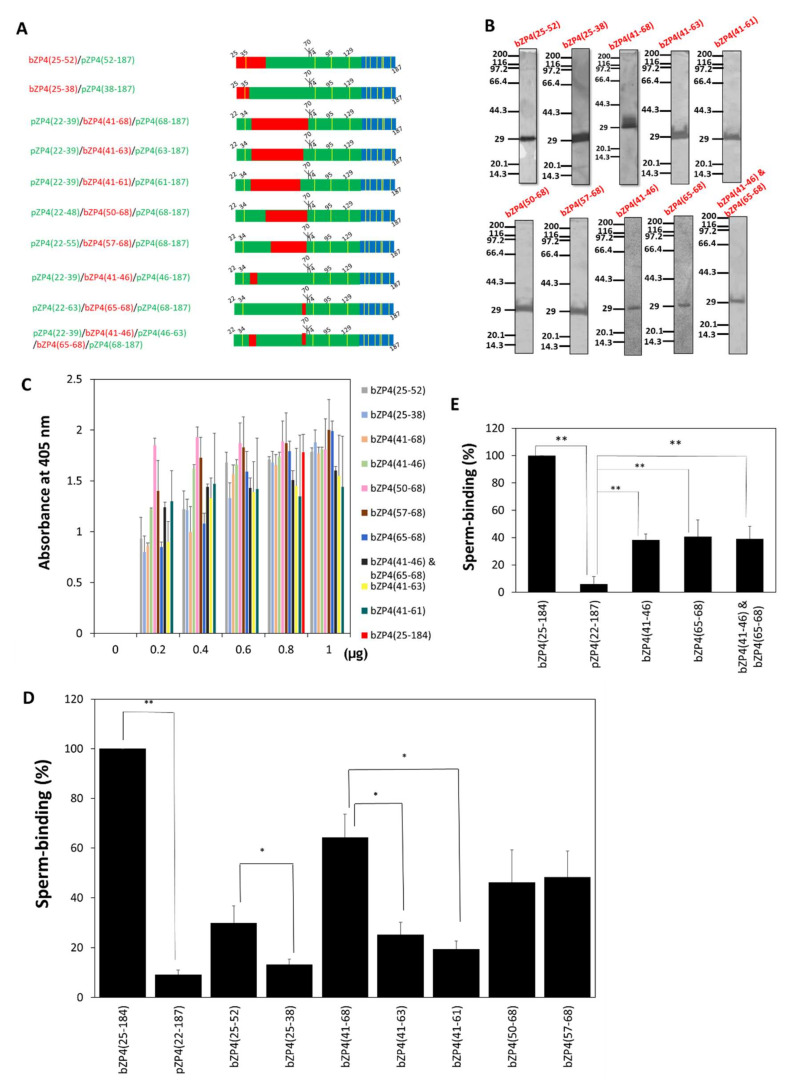
Two sites in the N-terminal half of the ZP-N1 domain of bZP4 are involved in the sperm-binding activity. (**A**) Schematic of bovine (red) and porcine (green) chimeric proteins in which bovine ZP4 (25–52), ZP4 (25–38), ZP4 (41–68), ZP4 (41–63), ZP4 (41–61), ZP4 (50–68), ZP4 (57–68), ZP4(41–46), bZP4(65–68) and both ZP4 (41–46) and ZP4 (65–68) replace the corresponding porcine sequences. The trefoil domain from pZP4 is shown in blue and Cys residues are in yellow bars. (**B**) SDS-PAGE of chimeric ZP4 proteins shown in (**A**). The proteins are represented in (**B**–**E**) by the bovine sequence regions shown in red in (**A**). The chimeric proteins were expressed in Sf9 cells and purified by TALON resin specific to His-tag. Gels were silver-stained. Molecular mass standards (kDa) are indicated on the left side of each panel. (**C**) Adsorption of each chimeric ZP4 protein shown in (**A**) to plastic wells. The amount of each chimeric ZP4 protein added to one plastic well is indicated under each group of bars. As a standard, 0.8 μg of bZP4(25–184) was added to a well (red bar). The amounts of proteins necessary for adsorption saturation were examined by detecting the adsorbed proteins with an antibody specific to the N-terminal His-tag. The experiment was performed three times and the average ± standard deviation (SD) of absorbance at 405 nm is shown. (**D**,**E**) Sperm-binding activity of each chimeric ZP4 protein shown in (**A**). Plastic wells were coated with each chimeric ZP4 protein (0.8 μg for each protein) indicated in the graph. The number of sperm bound to the wells coated with bZP4(25–184) varied from 33 to 65 but was designated as 100% for each experiment. Assays were repeated at least four times. Data are presented as the mean ± SD, with statistical significance between two bars indicated as *p* < 0.05 (*) and *p* < 0.01 (**) on each line connecting two bars.

Given that bZP4(25–135) (ZP-N1 domain of bZP4) is a sperm-binding region in bZP4 and that the trefoil domain (136–184) is not necessary for sperm binding [11], we investigated two bZP4(25–135) regions: bZP4(25–135) N-terminal (residues 25 to 73) and C-terminal (residues 74 to 135) halves. We first examined the sperm-binding sites in the N-terminal half of bZP4(25–135) considering the residues that were not conserved between bovines and pigs. Using pZP4(22–187) as a backbone, the porcine sequence was systematically replaced by the bovine sequence to express bZP4(25–52), bZP4(25–38), bZP4(41–68), bZP4(41–63), bZP4(41–61), bZP4(50–68) and bZP4(57–68) instead of the corresponding porcine sequences (Figure 3A). The chimeric fragments were purified to near homogeneity (Figure 3B) and were adsorbed to plastic wells (Figure 3C). The sperm-binding activity of bZP4(25–38)/pZP4(38–187) was not significantly different from that of pZP4(22–187) (Figure 3D), suggesting that the region from residues 25 to 38 is not necessary for bZP4(25–184) sperm-binding activity. The highest sperm-binding activity was shown by pZP4(22–39)/bZP4(41–68)/pZP4(68–187) and was similar to that of bZP4(25–73)/pZP4(73–187) (Figure 2D and Figure 3D). Considering that amino acid residues 39, 40 and 69 to 73 are identical between bovines and pigs (Figure S1), this result suggested that the region comprising residues 41 to 68 is a primary sperm-binding region in the ZP-N1 domain N-terminal half (25–73). 

The sperm-binding sites in this region (41–68) were investigated in more detail by comparing the sperm-binding activities of chimeric recombinant proteins. The sperm-binding activity of bZP4(25–52)/pZP4(52–187) was significantly higher than that of bZP4(25–38)/pZP4(38–187) (Figure 3D). Considering that the residues 39 and 40 are conserved between bovines and pigs, this result indicated that the region comprising residues 41 to 52 is involved in the sperm-binding activity of bZP4(25–184). The difference in sperm-binding activities between pZP4(22–48)/bZP4(50–68)/pZP4(68–187) and pZP4(22–55)/bZP4(57–68)/pZP4(68–187) was not significant, suggesting that the residues 50 to 56 were not important for the sperm-binding activity of bZP4(25–184) (Figure 3D and Figure S1). Therefore, the region extending from residues 41 to 49 was likely involved in sperm binding.

The difference in sperm-binding activities between pZP4(22–39)/bZP4(41–61)/pZP4(61–187) and pZP4(22–39)/bZP4(41–63)/pZP4(63–187) was not significant, suggesting that the residues 62 and 63 were not important for sperm-binding activity. The binding of pZP4(22–39)/bZP4(41–63)/pZP4(63–187) was significantly lower than that of pZP4(22–39)/bZP4(41–68)/pZP4(68–187), implying that the residues 64 to 68 were involved in sperm binding.

The binding of pZP4(22–48)/bZP4(50–68)/pZP4(68–187) was a little lower than that of pZP4(22–39)/bZP4(41–68)/pZP4(68–187), but the difference was not significant (Figure 3D). Therefore, the regions extending from residues 41 to 49 and from 64 to 68 of the N-terminal half (25–73) of the ZP-N1 domain were likely involved in sperm binding, albeit with the contribution of residues 41 to 49 being lower. 

We made further chimeric proteins considering that the residues 47 to 49 and 64 are conserved between bovines and pigs (Figure S1). Using pZP4(22–187) as a backbone, the porcine sequence was replaced by the bovine sequence to express bZP4(41–46), bZP4(65–68) and both bZP4(41–46) and bZP4(65–68) instead of the corresponding porcine sequences (Figure 3A). The chimeric fragments were purified to near homogeneity (Figure 3B) and were adsorbed to plastic wells (Figure 3C). The sperm-binding activities of chimeric proteins containing bZP4(41–46), bZP4(65–68) or both bZP4(41–46) and bZP4(65–68) were comparable and significantly higher than that of pZP4(22–187) (Figure 3E), whereas they were similar to that of bZP4(25–73)/pZP4(73–187) (Figure 2D and Figure 3E), suggesting that the residues 41 to 46 (site I) and 65 to 68 (site II) were involved in bZP4(25–184) sperm-binding activity.

#### 2.3.3. Identification of Sperm-Binding Regions in the C-Terminal Half of the ZP-N1 Domain

**Figure 4:**
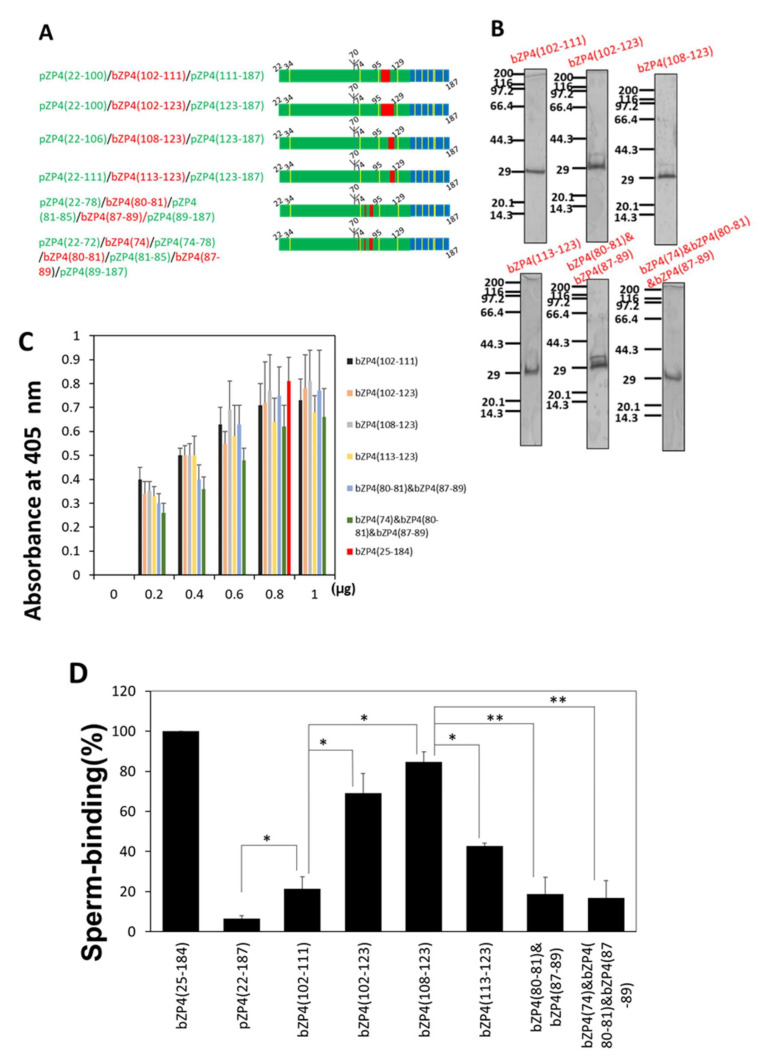
Sperm-binding sites in the C-terminal half of the ZP-N1 domain of bZP4. (**A**) Schematic of bovine (red) and porcine (green) chimeric proteins in which bovine ZP4 (102–111), ZP4 (102–123), ZP4 (108–123), ZP4 (113–123), both of ZP4 (80–81) and ZP4 (87–89), and both of ZP4 (80–81) and ZP4 (87–89) with ZP4 (74) replace the corresponding porcine sequences. The trefoil domain derived from pZP4 is shown in blue and Cys residues are in yellow bars. (**B**) SDS-PAGE of each chimeric ZP4 protein shown in (**A**). The proteins in (**B**–**D**) are indicated by the bovine sequence region shown in red in (**A**). The chimeric proteins were expressed in Sf9 cells and purified by TALON resin specific to His-tag. Gels were silver-stained. Molecular mass standards (kDa) are indicated on the left side of each panel. (**C**) Adsorption of each chimeric ZP4 protein shown in (**A**) to plastic wells. The amount of each chimeric ZP4 protein added to one plastic well is indicated under each group of bars. As a control, 0.8 μg of bZP4(25–184) was added to a well (red bar). The amounts of proteins necessary for adsorption saturation were examined by detecting the adsorbed proteins with an antibody specific to the N-terminal His-tag. The experiment was performed three times and the average ± standard deviation (SD) of absorbance at 405 nm is shown. (**D**) Sperm-binding activity of each chimeric ZP4 protein shown in (**A**). Plastic wells were coated with each chimeric ZP4 protein (0.8 μg for each protein). The number of sperm bound to wells coated with bZP4(25–184) varied from 33 to 65 but was designated as 100% for each experiment. Assays were repeated at least three times. Data are presented as the mean ± SD, with statistical significance between two bars indicated as *p* < 0.05 (*) and *p* < 0.01 (**) on each line connecting two bars.

We investigated the sperm-binding sites in the bZP4(25–135) C-terminal half. As above, residues with the lesser homology between bovine and porcine sequences were chosen. Recombinant proteins consisting of chimeric bovine and porcine ZP-N1 domains were constructed using pZP4(22–187) as a backbone and replacing porcine sequences with bovine sequences to express bZP4(102–111), bZP4(102–123), bZP4(108–123), bZP4(113–123), both bZP4(80–81) and bZP4(87–89), or bZP4(74) in addition to bZP4(80–81) and bZP4(87–89) instead of the corresponding porcine sequences (Figure 4A). The chimeric fragments were purified to near homogeneity (Figure 4B) and adsorbed to plastic wells (Figure 4C). The number of bovine sperm bound to wells coated with pZP4(22–106)/bZP4(108–123)/pZP4(123–187) was the highest (Figure 4D), indicating that the residues 108 to 123 (site III) are necessary for the sperm-binding activity of the bZP4 ZP-N1 domain. 

The sperm-binding activity of pZP4(22–100)/bZP4(102–123)/pZP4(123–187) was not significantly different from that of pZP4(22–106)/bZP4(108–123)/pZP4(123–187) (Figure 4D), suggesting that the residues 102 to 107 were not necessary for binding sperm. The sperm-binding activity of pZP4(22–111)/bZP4(113–123)/pZP4(123–187) was significantly lower than that of pZP4(22–106)/bZP4(108–123)/pZP4(123–187) (Figure 4D), suggesting that the residues 108 to 112 were involved in the sperm binding. The sperm-binding activity of pZP4(22–100)/bZP4(102–111)/pZP4(111–187) was significantly lower than that of pZP4(22–100)/bZP4(102–123)/pZP4(123–187) (Figure 4D), suggesting the residues 112 to 123 were involved in the sperm binding. The sperm-binding activity of pZP4(22–100)/bZP4(102–111)/pZP4(111–187) was significantly higher than that of pZP4(22–187) (Figure 4D). Since pZP4(22–100)/bZP4(102–111)/pZP4(111–187) contained the residues 108 to 111 of the bovine sequence and considering that Glu-112 is conserved between bovines and pigs, these results suggested that the residues 108 to 111, i.e., site III N-terminal part, and the residues 113 to 123, i.e., site III C-terminal part, contributed to the sperm-binding activity of the ZP-N1 domain. 

The sperm-binding activities of pZP4(22–78)/bZP4(80–81)/pZP4(81–85)/bZP4(87–89)/pZP4(89–187) and pZP4(22–72)/bZP4(74)/pZP4(74–78)/bZP4(80–81)/pZP4(81–85)/bZP4(87–89)/pZP4(89–187) were much lower than that of pZP4(22–106)/bZP4(108–123)/pZP4(123–187) and were not significantly different from that of pZP4(22–187) (Figure 4D). Because six residues at positions 74, 80, 81 and 87 to 89 in the 74 to 93 region of the bovine sequence are not identical to the corresponding residues in the porcine sequence, this result suggested that the residues 74 to 93 were not involved in the sperm-binding activity of the ZP-N1 domain. An unknown technical problem prevented the generation of a plasmid with the Ser-94 to Arg mutation, thus, the involvement of Ser-94 in the sperm-binding activity was not clarified. Taken together, the results strongly suggested that the region extending from residues 108 to 123 (site III) is a sperm-binding site of the ZP-N1 domain of bZP4.

**Figure 5:**
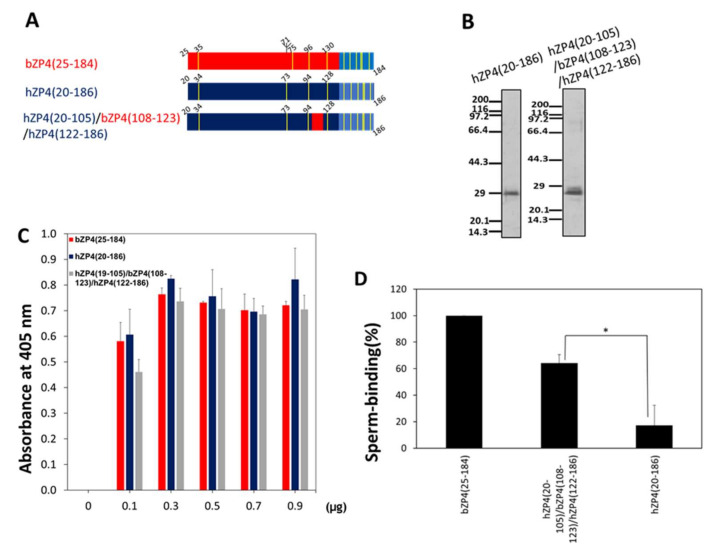
Sperm-binding activity of the sperm-binding site in the C-terminal half of the ZP-N1 domain of bZP4 in the framework of human ZP4. (**A**) Schematic of the truncated bZP4(25–184) (red), its corresponding human (h)ZP4(20–186) counterpart (dark blue) and the chimeric protein in which bZP4 (108–123) replaces the corresponding human sequence. Trefoil domains are shown in blue and Cys residues are in yellow bars. (**B**) SDS-PAGE of hZP4(20–186) and the bovine/human chimeric ZP4 protein, bZP4(20–105)/bZP4(108–123)/hZP4(122–186). The proteins were expressed in Sf9 cells and purified by TALON resin specific to His-tag. Gels were silver-stained. Molecular mass standards (kDa) are indicated on the left side of each panel. (**C**) Adsorption of bZP4(25–184) (red), hZP4(20–186) (dark blue) and bZP4(20–105)/bZP4(108–123)/hZP4(122–186) (gray) to plastic wells. The amount of each ZP4 fragment added to one plastic well is indicated under each group of bars. The amounts of proteins necessary for adsorption saturation were examined by detecting the adsorbed proteins with an antibody specific to the N-terminal His-tag. The experiment was performed three times and the average ± standard deviation (SD) of absorbance at 405 nm is shown. (**D**) Sperm-binding activities of the ZP4 fragments shown in (**A**). Plastic wells were coated with each ZP4 protein (0.8 μg for each protein) indicated in the graph. The number of sperm bound to the wells coated with bZP4(25–184) varied from 33 to 65 but was designated as 100% for each experiment. Assays were repeated at least three times. Data are presented as the mean ± SD, with statistical significance between the chimeric fragment and hZP4(20–186) indicated *p* < 0.05 (*) on the line connecting two bars.

To further confirm the involvement of site III of the C-terminal region of bZP4(25–135) in sperm binding, we prepared a chimeric bovine and human protein in which bZP4(108–123) replaced the corresponding hZP4(20–186) sequence (Figure 5A and Figure S1). The hZP4(20–186) and chimeric hZP4(20–105)/bZP4(108–123)/hZP4(122–186) fragments were expressed in Sf9 cells, purified (Figure 5B) and adsorbed to plastic wells (Figure 5C). Bovine sperm failed to recognise hZP4(20–186) (Figure 5D). Remarkably, the sperm-binding activity of the chimeric hZP4(20–105)/bZP4(108–123)/hZP4(122–186) protein was significantly increased compared with that of hZP4(20–186) (Figure 5D). Collectively, solid support assays using bovine/porcine and bovine/human chimeric proteins revealed that three sites, i.e., site I: bZP4(41–46), site II: bZP4(65–68) and site III: bZP4(108–123), of the ZP-N1 domain of bZP4 play significant roles in bovine sperm recognition.

## 3. Discussion

The structures and functions of the three proteins (ZP1, ZP2 and ZP3) present in mouse ZP have been studied extensively. ZP2 and ZP3 are the main ZP glycoproteins [7] and are involved in the interaction with sperm [24]. The less abundant ZP1 is not directly involved in sperm binding but covalently crosslinks ZP filaments to maintain the structural integrity of the ZP matrix [25,26]. Although human ZP1, ZP2 and ZP3 biological activities are similar to those of the corresponding mouse ZP glycoproteins [27], the lack of ZP4 in the mouse has hindered the study of the protein and its biological function remains unknown.

To evaluate the role of individual bovine ZP proteins in sperm recognition, we previously developed a solid support assay by coating plastic plates with individual ZP proteins and found that ZP2 and ZP3 are not involved in sperm binding, whereas ZP4 multimerisation is important for the sperm-binding activity [11]. We also identified the N-terminal ZP-N1 domain of ZP4 as responsible for the sperm-binding activity of the protein [11]. The present study aimed to decipher the sperm-ZP binding mechanism in detail. We took advantage of the solid support assay system to analyse sperm-binding sites in the ZP-N1 domain.

**Figure 6:**
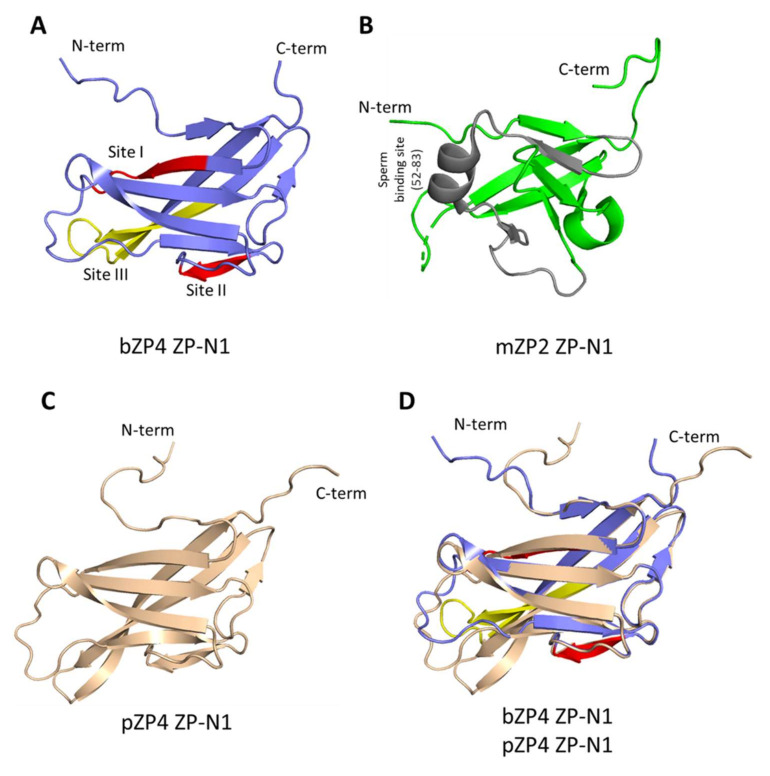
Structural comparison among N-terminal ZP-N1 domains of mouse (m)ZP2, bovine (b)ZP4 and porcine (p)ZP4. (**A**) A predicted structural model of the ZP-N1 domain of bZP4. The model was made using AlphaFold2 [28]. Three sperm-binding sites proposed in the present study are highlighted in red (Sites I and II) and in yellow (Site III). (**B**) Crystal structure of the N-terminal ZP-N1 domain of mZP2 (PDB 5II6) [27]. Mouse sperm-binding region is highlighted in gray. (**C**) A predicted structural model of the ZP-N1 domain of pZP4. The model was made using AlphaFold2 [28]. (**D**) Superimposition of the predicted 3D models of the ZP-N1 domains of bZP4 and pZP4.

We found that bZP4(25–184) sperm-binding activity was independent of Asn-71 N-glycosylation, as mutation of the site did not change sperm-binding activity. The sperm-binding activity is species-selective, as bovine sperm failed to recognise both pZP4(22–187) and hZP4(20–186). We further utilised this species-selective sperm recognition and by analysing bovine/porcine and bovine/human chimeric proteins, identified three sites, site I: bZP4(41–46), site II: bZP4(65–68) and site III: bZP4(108–123), in the N-terminal ZP-N1 domain of bZP4(25–135), that are important for sperm-binding activity in bovines. No crystal structure of the N-terminal ZP-N1 domain of bZP4(25–135) is yet available. However, we generated a 3D model of bZP4(25–135) (Figure 6A).

A single domain in the ZP2 N-terminus is needed for human and mouse sperm recognition [8,9]. In humans, the region extending from residues 56 to 87, namely between Cys-55 and Cys-88, of hZP2 was identified as a sperm-binding site by using chimeric human/mouse recombinant proteins of hZP2(39–154) [9]. In mice, the corresponding residues from 52 to 83 in the N-terminal domain of mZP2 were suggested to be a sperm-binding site [9]. Recently, the crystal structure of the mZP2 N-terminal domain, mZP2(35–138) was obtained [27] (Figure 6B). The 3D model of bZP4(25–135) and the crystal structure of mZP2(35–138) are similar, and their β-strand cores make two antiparallel β-sheets (Figure 6A,B). Importantly, the residues 41 to 68, including bZP4(25–135) sites I and II, almost overlap with the 52 to 83 region of mZP2. This suggests that the sperm-binding regions of the ZP-N1 domain of bZP4 and of the mZP2 N-terminal domain overlap. A notable difference is that site III is involved in bZP4(25–135) sperm binding in addition to sites I and II. The site III corresponds to the loop region comprising residues 116 to 130 in the mZP2 sequence which connects the two antiparallel β-strands F and G in mZP2(35–138) (Figure 6B) [27]. In the crystal structure of mZP2(35–138), residues 52 to 83 and the loop region (residues 116 to 130) are close to each other and seem to form an interaction surface (Figure 6B) [27]. Since the involvement of the 116 to 130 region of mZP2 or of the corresponding hZP2 region in sperm-binding has not been reported, the role of bZP4(25–135) site III in sperm-binding is, so far, unique to the ZP-N-like domain of bZP.

The difference in the sperm-binding activities between pZP4(22–39)/bZP4(41–61)/pZP4(61–187) and pZP4(22–39)/bZP4(41–63)/pZP4(63–187) was not significant (Figure 3D) and the difference in the sperm-binding activities between pZP4(22–48)/bZP4(50–68)/pZP4(68–187) and pZP4(22–55)/bZP4(57–68)/pZP4(68–187) was not significant (Figure 3D). These results suggested that the residues 50 to 56, 62 and 63, which are located between site I and site II, were not important for the sperm-binding activity of bZP4(25–184). The 3D model of bZP4(25–135) proposes a hypothesis that the loop region between site I and site II is involved in sperm binding, but the results in this study did not support this hypothesis. We made a 3D model of the N-terminal ZP-N1 domain of pZP4, pZP4(22–136) (Figure 6C). The 3D models of the ZP-N1 domains of bZP4 and pZP4 are very similar and could be superimposed (Figure 6D). The probabilities of the 3D model prediction in the loop areas on the left side of the β-strand cores were low for both bZP4 and pZP4. So, it is not yet possible to discuss the molecular mechanisms of the species-selective recognition of the bovine and porcine ZP-N1 domains by bovine sperm based on the 3D models. 

Both site I and site II contain Pro at positions 46 and 66, respectively, which are not conserved in the porcine sequence. This raises the possibility that the Pro residues are directly or indirectly involved in bZP4(25–135) sperm-binding activity. However, this remains to be clarified. The identity between site III of bZP4 and the corresponding region of pZP4 is 25%, whereas the identity between the other region of bZP4(25–135) and the corresponding region of pZP4 is 63%. This suggests that the unique structural elements of bZP4(25–135) site III are important for species-selective sperm recognition. 

ZP1 and ZP4 share sequence similarities and present a similar domain organisation [29]. It is believed that they have arisen from a common gene after a gene duplication event that occurred after the fish lineage [30]. Considering that human sperm do not recognise mouse ZP, ZP4 was initially proposed as a sperm ligand in humans. However, this possibility was not supported by the study of transgenic animals expressing mZP1–3 and hZP4, as hZP4 failed to bind human sperm [31]. In addition, sperm binding is normal in ZP4-knock out rabbits [32]. ZP4 is also dispensable for rat fertilisation [33]. However, recent work has suggested that ZP4 functions diverge amongst vertebrates [34]. The ZP4 biological role in species possessing ZP1 might be different from that in bovines which lack ZP1. Although the findings of the present in vitro biochemical study suggest that in bovines ZP4 plays a role in sperm recognition, sperm factors recognising the sperm-binding sites in bZP4(25–135) remain to be identified and further in vivo validation of these observations in cow fertilisation is necessary.

## 4. Materials and Methods

### 4.1. Expression and Purification of Recombinant ZP Proteins

Translational Met was numbered as 1 for bZP4, pZP4 and hZP4 fragments. There were gaps of amino acids in the alignment of bovine, porcine and human amino acid sequences. Therefore, the numbering of corresponding residues did not match amongst the three fragments (Figure S1).

#### 4.1.1. Construction of Recombinant Baculovirus Transfer Plasmids Encoding ZP Proteins

The construction of the pBACgus6 transfer plasmid encoding N-terminally His-tagged (His-His-His-His-His-His-) and S-tagged (Lys-Glu-Thr-Ala-Ala-Lys-Phe-Glu-Arg-Gln-His-Met-Asp-Ser-) bZP4(25–184) was reported previously [11]. The preparation of pBACgus6 plasmids encoding bZP4(25–184) N71Q was performed with the PrimeSTAR Mutagenesis Basal Kit (Takara, Kyoto, Japan) using the bZP4(25–184) plasmid as a template. The cDNA encoding pZP4 (22–187) was commercially synthesised (Integrated DNA technologies, Tokyo, Japan). This fragment was inserted into EcoRI/HindIII sites of pBACgus6. There is a unique PstI site at the corresponding sites of pBACgus6-bZP4(25–184) and pBACgus6-pZP4(22–187). The preparation of the pBACgus6 plasmid encoding bZP4(25–73)/pZP4(73–187) was performed by ligating the PstI/HindIII fragment of pBACgus6-pZP4(22–187) to the pBACgus6-bZP4(25–184) plasmid digested with PstI/HindIII. The pBACgus6 plasmid encoding pZP4(22–67)/bZP4(69–184) was obtained by ligating the PstI/HindIII fragment of pBAgus6-bZP4(25–184) to the pBACgus6-pZP4(25–187) plasmid digested with PstI/HindIII. Plasmids encoding more detailed chimeric proteins in the bZP4(25–73) region were prepared by successively changing bovine nucleotide sequences to porcine sequences with the PrimeSTAR Mutagenesis Basal Kit (Takara) using the plasmid encoding bZP4(25–73)/pZP4(73–187) as a template. Plasmids encoding more detailed chimeric proteins in the bZP4(69–135) region were prepared by successively changing porcine nucleotide sequences to bovine sequences with the PrimeSTAR Mutagenesis Basal Kit (Takara) using the plasmid encoding pZP4(22–187) as a template. The cDNAs encoding hZP4(20–186) and hZP4(20–105)/bZP4(108–123)/hZP4(122–186) were commercially synthesised (Integrated DNA technologies). These fragments were ligated to pBACgus6 using an in vivo *E. coli* cloning system, iVEC [35]. The DNA sequences of the constructed plasmids were confirmed using a commercial DNA sequencing service (Macrogen, Fukuoka, Japan).

#### 4.1.2. Expression of Recombinant ZP Proteins

The preparation of recombinant viruses was performed according to the previous report [11]. Sf9 cells were routinely propagated in Sf-900II serum-free medium (Invitrogen, Carlsbad, CA, USA). Each of the constructed baculovirus transfer plasmids was transfected along with flashBAC DNA (Oxford Expression Technologies, Oxford, UK) into Sf9 insect cells according to the manufacturer’s protocol. Sf9 cells were infected with each recombinant virus, and the expression and secretion of each recombinant protein into the culture supernatant were verified as previously reported [11]. All recombinant ZP proteins were expressed as secretory proteins using a signal peptide derived from pBACgus6.

#### 4.1.3. Purification of Recombinant ZP Proteins from Culture Supernatants

Purification of recombinant ZP proteins was also performed according to the previous report [11]. Briefly, for large-scale protein expression, 200 mL of Sf9 cells (1.0 × 10^6^ cells/mL) were infected with each recombinant virus and cultured for 48 h at 27 °C in suspension. Each recombinant protein was purified with TALON metal affinity resin^®^ (Takara) using the N-terminal His-tag of the proteins. 

#### 4.1.4. SDS-PAGE

SDS-PAGE was performed on 12.5% (*w*/*v*) separating gels under reducing conditions according to the Laemmli method [36]. The gels were silver-stained. Standard proteins with a broad molecular mass range (Takara) were used to estimate apparent protein molecular masses. A prestained molecular mass marker (SMOBIO Tech, Hsinchu City, Taiwan) was used for Western blots.

### 4.2. Immunoblot Analysis

SDS-PAGE resolved proteins were processed for immunoblot analysis. After transfer of resolved proteins to Immobilon-P membranes (Millipore, Bedford, MA, USA), the membranes were blocked with 3% bovine serum albumin (BSA) in Tris-buffered saline (TBS) for 1 h at room temperature, rinsed once with TBS and incubated with anti-His-tag antibody (Wako, Kyoto, Japan) 3000-fold diluted with TBS containing 1% BSA for 2 h. The membranes were then washed three times for 15 min each with TBS containing 0.05% Tween 20 (T-TBS) and then incubated for 1.5 h with horseradish peroxidase (HRP)-conjugated rabbit anti-mouse IgG (Wako) 1000-fold diluted with TBS containing 1% BSA. After washing three times for 15 min each with T-TBS, the blots were developed using 3,3′,5,5′-tetramethyl benzidine (TMB) (SeraCare, Gaithersburg, MD, USA).

### 4.3. Lectin Blot Analysis

Alternatively, SDS-PAGE resolved proteins were transferred to Immobilon-P membranes. The membranes were blocked with TBS containing 3% BSA for 1 h and then incubated for 2 h with 1 µg/mL of biotin-conjugated GNA (EY Laboratories, San Mateo, CA, USA) in T-TBS. Membranes were washed three times for 15 min each with T-TBS and incubated for 1 h with 0.5 µg/mL of HRP-conjugated streptavidin (Sigma-Aldrich) in T-TBS. Membranes were then washed three times for 15 min each with T-TBS, followed by color development using TMB.

### 4.4. Measurement of the Sperm-Binding Activity of Recombinant ZP Proteins by the Solid Support Assay

This procedure was performed according to the previous report [11] as described briefly below.

#### 4.4.1. Adsorption of Recombinant ZP Proteins to Plastic Wells

The amount of each recombinant ZP protein that was enough for saturated adsorption to a well was investigated by adding 50 μL of protein solution at different concentrations to a 96-well plate (Nalge Nunc, Rochester, NY, USA) and incubating the plate overnight at 4 °C. As a control, 0.8 μg of bZP4 was added to a well. The wells were rinsed with phosphate-buffered saline (PBS: 40 mM KH_2_PO_4_, 150 mM NaCl, pH 7.4). The wells were blocked with 3% BSA in TBS for 1 h at room temperature. After washing the wells three times with TBS, an antibody against His-tag (Wako) 3000-fold diluted with TBS containing 1% BSA was added to each well and incubated for 1 h at room temperature. The wells were washed three times with T-TBS and incubated with HRP-conjugated rabbit anti-mouse antibody (Wako) 1000-fold diluted with TBS containing 1% BSA. After washing three times with T-TBS, 2,2′-Azinobis (3-ethylbenzothiazolin-6-sulfonic acid) (ABTS; Roche) was added to each well as a substrate of HRP. After incubating for 1 h at room temperature, absorbance at 405 nm was measured with a plate reader (TECAN, Mannedorf, Swiss).

#### 4.4.2. Sperm Binding to Recombinant ZP Proteins Adsorbed to Plastic Wells

The designated amount of each protein was added to a 96-well plate (Nalge Nunc) and incubated overnight at 4 °C. As a negative control, 50 μL of elution buffer alone, used for TALON column chromatography, was adsorbed. The solution in the wells were discarded, and the wells were washed once with PBS and then blocked with 3% BSA in TBS at 38.5 °C for 2 h. The frozen Holstein bull sperm straws supplied for artificial insemination were purchased from Animal Genetics Japan Co., Ltd. (Matsuzaka, Japan). Frozen bovine sperm was thawed and washed twice in pre-warmed (38.5 °C) Brackett and Oliphant (BO) solution without BSA [11,37]. The bovine sperm were then capacitated by incubation in BO solution containing BSA for 30 min. Capacitation and subsequent incubations were carried out at 38.5 °C under 2% CO_2_. Aliquots (50 μL) containing 4 × 10^5^ capacitated sperm were transferred into the wells, and the plates were incubated for 2 h. The wells were washed three times with BO solution and then 50 μL of 70% glycerol in PBS was added to each well, and the sperm bound to the wells were recovered by 20 strokes of vigorous pipetting. The number of sperm in 0.1 μL of suspension was determined using a haemocytometer. The number of sperm bound to the wells not coated with recombinant ZP proteins (0 to 5) was subtracted from the number of sperm bound to the wells coated with recombinant ZP proteins. The average number of sperm in 0.1 μL of suspension in the 100% sperm-binding control of experiments is shown in the legends for figures.

#### 4.4.3. Statistical Analysis

Welch’s t-test was used to determine whether there was a significant difference in the sperm count between two groups. Differences were considered to be significant at *p* < 0.05.

## Data Availability

Raw data is available on request from the corresponding author (N.Y.).

## Supplementary Materials

The following supporting information can be downloaded at: https://www.mdpi.com/article/10.3390/ijms23020762/s1.
