# Supplementary material for: Identification of Sperm-Binding Sites in the N-Terminal Domain of Bovine Egg Coat Glycoprotein ZP4
Source: Int J Mol Sci. 2022 Jan 11;23(2):762. doi: 10.3390/ijms23020762 (PMC8775842; doi:10.3390/ijms23020762)
Supplement: Supplementary file 1 [file ijms-23-00762-s001.zip › ijms-1530844-supplementary.pdf]

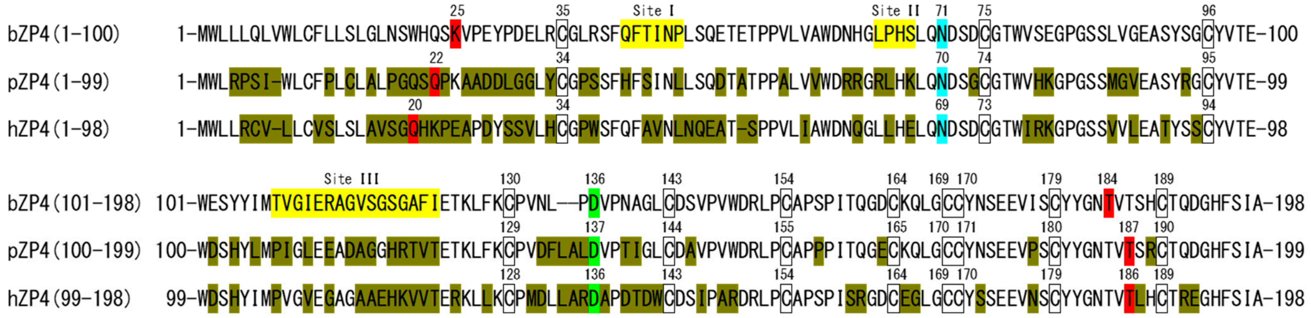

Figure S1. Amino acid sequence alignment of ZP4 among bovines, pigs and humans. Amino acid sequences from translational initiation Met (as number 1) to the N-terminal part of ZP module are aligned among bovine (b)ZP4 (1-199), porcine (p)ZP4(1-198) and human (h)ZP4(1-198). N-terminal residues of ZP4 following N-terminal tags derived from pBACgus transfer vector and C-terminal residues of the recombinant fragments expressed in this study are shown in red: Lys-25 and Thr-184 in bovine fragment; Gln-22 and Thr-187 in porcine counterpart; Gln-20 and Thr-186 in human counterpart. N-glycosylation site, Asn-71 in bovine fragment and the corresponding residues in its porcine and human counterparts are highlighted in cyan. The beginning of trefoil domain, Asp-136 in bovine fragment and the corresponding residues in its porcine and human counterparts are highlighted in green. Cys residues in the mature polypeptides are all conserved and highlighted by boxes. The amino acid residues in porcine and human sequences not identical to the corresponding residues in bovine sequence are highlighted in gold. Sperm-binding sites (site I, site II and site III) in bovine fragment identified in this study are highlighted in yellow.
